# Plumbing Pathogens: A Fixture in Hospitals and Homes

**DOI:** 10.1289/ehp.123-A217

**Published:** 2015-08-01

**Authors:** Carol Potera

**Affiliations:** Carol Potera, based in Montana, also writes for *Microbe*, *Genetic Engineering News*, and the *American Journal of Nursing*.

Practicing good hygiene is supposed to make you healthier, not sicker. However, a growing body of research shows that certain bacteria can thrive in household and hospital plumbing systems and may cause life-threatening infections among susceptible individuals after inhalation or ingestion. In this issue of *EHP*, Joseph Falkinham of Virginia Polytechnic Institute and State University in Blacksburg and colleagues review the epidemiology and ecology of what are known as opportunistic premise plumbing pathogens (OPPPs).^1^

“Premise plumbing” refers to the pipes and fixtures within a building that transport water to taps after it is delivered by the utility. OPPPs are so ubiquitous in plumbing systems that many experts now consider them normal inhabitants, rather than contaminants, of drinking water distribution systems. The new review focuses on *Legionella pneumophila*, *Mycobacterium avium*, and *Pseudomonas aeruginosa*, three of the best studied OPPPs.

**Figure:**
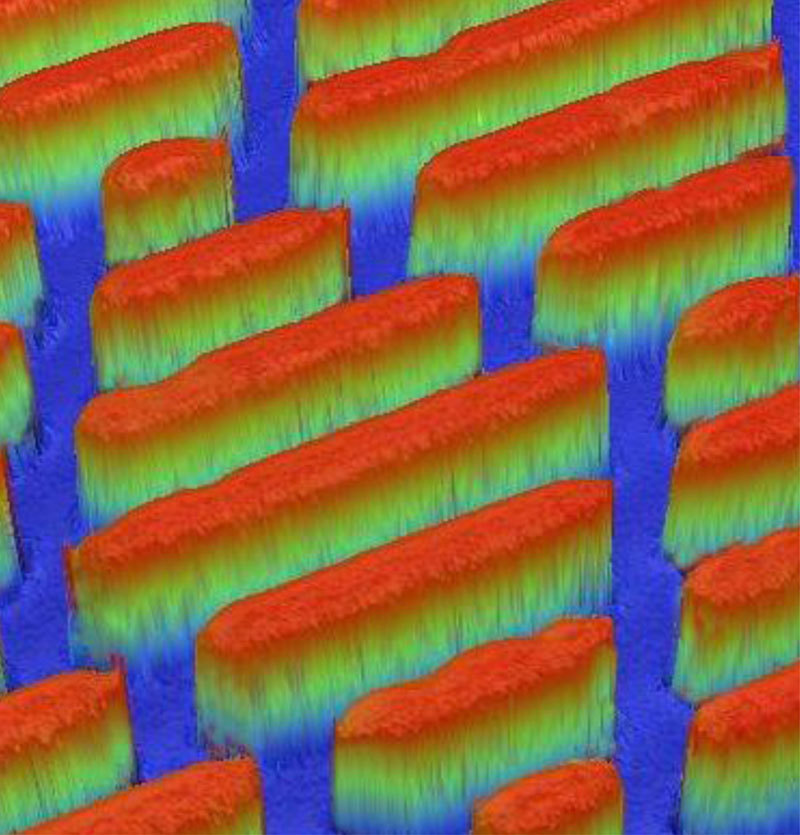
Sharklet’s micropatterned surface deters the formation of biofilms without the use of chemical antimicrobials. Materials such as this may be one way to keep OPPPs from colonizing plumbing systems. Source: Mann et al. (2014)^7^

OPPPs are estimated to cause nearly 30,000 cases of human disease yearly at a cost of $850 million.^2^ Elena Naumova, director of the Tufts University Initiative for the Forecasting and Modeling of Infectious Disease, says this is likely a substantial underestimate, because these pathogens are rarely tested for in clinical settings, even in severely ill patients.

In addition, Naumova says, the clinical manifestations of OPPP-related diseases often include common symptoms such as high fever, chills, and fatigue, making it difficult to distinguish them from other pneumonic infections. “The need for identification and reporting these infections is an important conclusion of the review,” she says. Naumova was not involved with the review.

Other waterborne pathogens, such as poliovirus and Salmonella, are more readily killed by disinfectants, and they generally do not reproduce in plumbing systems. In contrast, OPPPs attach to pipe surfaces and grow as recalcitrant biofilms in low-nutrient, stagnant water. They are killed by neither common disinfectants nor natural predators, such as amoebae. Instead, OPPPs multiply inside amoebae after ingestion. “OPPPs are perfectly adapted to drinking water systems,” says Falkinham.

The three OPPPs reviewed by Falkinham and his colleagues are particularly problematic in premise plumbing, and practices for control of these organisms are not well validated, says Mark LeChevallier, director of innovation and environmental stewardship at the New Jersey–based utility American Water. LeChevallier says the authors’ recommendations on research needs will “improve our understanding of the epidemiology and ecology of these emerging pathogens.”

One proposed control method for OPPPs is simply to raise the temperature of hot water systems. In a small ongoing study of patients infected with *M. avium*, increasing the set point on home water heaters from the recommended 120°F to 140°F appears helpful in eliminating *M. avium* in home plumbing systems. However, the unpublished study includes just 10 homes, and it’s too early to issue a general recommendation. “We worry about people scalding themselves, and it counteracts energy company pleas to lower temperatures to conserve energy,” says Falkinham.

Some hospitals use a 0.2-µm microbiological filter, such as those made by Pall Medical, on showerheads and faucets in patient rooms to block OPPPs. Steve Ebersohl, senior director of hospital sales with Pall Medical, says physicians who treat OPPP patients can refer them to that company or other manufacturers to learn how to add these filters to their home plumbing fixtures. However, activated charcoal filters, such as the ones often used to purify home water supplies, do not block and may even increase the growth of OPPPs. *M. avium*, for instance, flourishes on activated charcoal filters,^3^ where it is supplemented by the trapped metals and organic matter, Falkinham says.

Another option is to coat pipe interiors with agents that block biofilm growth. Sharklet, a synthetic material that mimics the rough texture of natural shark skin, resists biofouling and reduces biofilm formation by *M. avium*.^4^ Made by Sharklet Technologies in Aurora, Colorado, this novel coating has microscopic ribs that discourage pathogens from settling on it. Sharklet Technologies is currently evaluating the material’s ability to prevent biofilm fouling in surgical and hospital settings.^5^

The common traits shared by *L. pneumophila*, *M. avium*, and *P. aeruginosa* suggest they could be controlled collectively by a few effective treatments, Falkinham says. He hopes those same solutions also may prevent widespread public health impacts associated with emerging OPPPs, such as multidrug-resistant *Acinetobacter*, which has infected soldiers who were wounded serving in the Middle East.^6^
